# Cell Wall Structure of Coccoid Green Algae as an Important Trade-Off Between Biotic Interference Mechanisms and Multidimensional Cell Growth

**DOI:** 10.3389/fmicb.2018.00719

**Published:** 2018-04-13

**Authors:** Susanne Dunker, Christian Wilhelm

**Affiliations:** ^1^Helmholtz-Centre for Environmental Research – UFZ, Leipzig, Germany; ^2^German Centre for Environmental Research – iDiv, Leipzig, Germany; ^3^Department of Plant Physiology, University of Leipzig, Leipzig, Germany

**Keywords:** algaenan, cell wall structure, *Microcystis aeruginosa*, interference interaction, green algae, trilaminar cell wall

## Abstract

Coccoid green algae can be divided in two groups based on their cell wall structure. One group has a highly chemical resistant cell wall (HR-cell wall) containing algaenan. The other group is more susceptible to chemicals (LR-cell wall – Low resistant cell wall). Algaenan is considered as important molecule to explain cell wall resistance. Interestingly, cell wall types (LR- and HR-cell wall) are not in accordance with the taxonomic classes Chlorophyceae and Trebouxiophyceae, which makes it even more interesting to consider the ecological function. It was already shown that algaenan helps to protect against virus, bacterial and fungal attack, but in this study we show for the first time that green algae with different cell wall properties show different sensitivity against interference competition with the cyanobacterium *Microcystis aeruginosa*. Based on previous work with co-cultures of *M. aeruginosa* and two green algae (*Acutodesmus obliquus* and *Oocystis marssonii*) differing in their cell wall structure, it was shown that *M. aeruginosa* could impair only the growth of the green algae if they belong to the LR-cell wall type. In this study it was shown that the sensitivity to biotic interference mechanism shows a more general pattern within coccoid green algae species depending on cell wall structure.

## Introduction

The importance of cell wall structure relies on the fact that the cell wall is the outer boundary of the cell that interacts directly with the external environment. Coccoid green algae have two different outer cell wall structure types with regard to chemical resistance (Burczyk et al., 1995). Burczyk et al. (2014) could clearly differentiate between taxa of low and high chemical resistance. HR-cell wall structures were found in several species of the genera *Chlorella, Scenedesmus*, and *Botryococcus* (Allard and Templier, 2000; Scholz et al., 2014). One important chemical structure of cell wall resistance is the biopolymer algaenan. Several different names exist for this biomolecule, like “sporopollenin” (Atkinson et al., 1972), PRB A and B (Polymère resistant de *Botryococcus* (Berkaloff et al., 1983) or ARB (acetolysis resistant biopolymer) (Burczyk and Loos, 1995). Algaenan is a polyester heteropolymer which is highly acid and base-resistant compound due to a steric protection effect of the molecular structure (Largeau and De Leeuw, 1995). It is sufficiently resistant that it is part of today‘s kerogen (Goth et al., 1988; Versteegh and Blokker, 2004). Kerogen is defined by Oilfield Glossary as naturally occurring organic matter that is non-extractable using organic solvents. Based on the extreme resistance of this molecule, chemical analysis of algaenan is quite challenging (Kodner et al., 2009).

In many cases algaenan is part of tri-laminar structure/sheath (TLS), where the cell wall consists of 10–20 nm thick sandwich-like layers with two outside layers of high and one inside layer with low electron density (Allard and Templier, 2000; Versteegh and Blokker, 2004; Burczyk et al., 2014). Coccoid green algae species which lack algaenan are characterized by having a homogenous, carotenoid-free outer cell wall layer (Burczyk and Loos, 1995). However, no rule without exceptions: tri-laminar cell wall and algaenan content are not necessarily coupled; a lack of algaenan was found in species containing TLS and vice versa (Largeau and De Leeuw, 1995). As additional structural components responsible for protective cell wall, acidic and hydrophobic pectins and also glycoprotein could come into question (Voigt et al., 2014).

The focus of this study relies on differential resistance of coccoid green algae, identified by literature research on presence/absence of algaenan, various staining procedures and derived from physiological measures. The authors do not aim to biochemically characterize the species in detail, but focus on potential ecological consequences of cell wall resistance.

During the last years an increasing interest on cell wall structures mainly arised from biotechnological research. Cell wall structure is a crucial factor for biotechnological extraction processes of intracellular compounds (Lee et al., 2012; Kim et al., 2013; Gerken et al., 2013; Burczyk et al., 2014; Baudelet et al., 2017). Much more energy is required for extraction of intracellular compounds from species with an algaenan containing cell wall (Lee et al., 2012). The genus *Botryococcus* is a common example, with promising high intracellular oil content per cell but an algaenan-containing cell wall (Kodner et al., 2009; Watanabe et al., 2014).

Biological investigations about cell wall structure of coccoid green algae are limited (Kodner et al., 2009). Interestingly, algaenan cannot be used as suitable biomarker for specific green algae, because algaenan-containing cell wall is not a specific feature of one chlorococcal class (Kodner et al., 2009; Baudelet et al., 2017), which makes it even more interesting to consider the ecological relevance of different cell wall structures within coccoid green algae. Due to different chemical properties of a highly resistant (HR) cell wall structure, the cell is highly protected against enzymatic and chemical attack (Versteegh and Blokker, 2004), e.g., by bacteria or fungi (Derenne et al., 1992; Largeau and De Leeuw, 1995). It was also proposed that nutrient-limited algae with thick algaenan-containing cell walls survive the gut passage by daphnid feeders (van Donk and Hessen, 1993; van Donk et al., 2011), because algaenan is resistant to gut enzymes.

**FIGURE 1:**
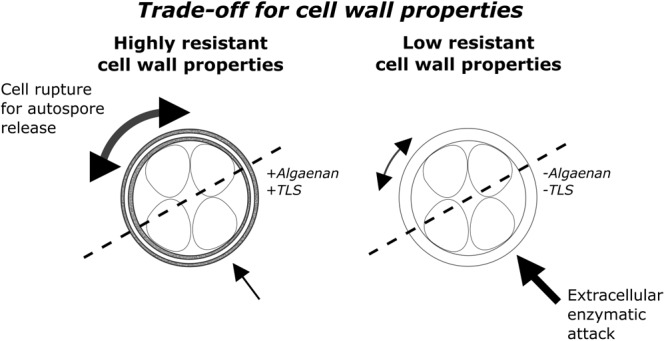
Conceptual visualization of the trade-off between enzymatic attack and multidimensional cellular growth as well as autospore release. On the left side a HR-cell wall structure type with a trilaminar algaenan-containing sheath and on the right side a LR-cell wall structure type with homogenous outer cell wall is shown.

The relevance of different cell wall structure for interspecific biotic interactions among phytoplankton species has not been examined in this context so far and should therefore be the aim of this study (**Figure 1**). In previous experiments with two co-cultivated green algae and the cyanobacterium *Microcystis aeruginosa* it was shown that the growth of the green algae *Oocystis marssonii*, which is not supposed to have an algaenan-containing cell wall, was strongly inhibited by co-cultivation with *M. aeruginosa*. In contrast, growth of *Acutodesmus obliquus*, which has an algaenan-containing cell wall, was not affected although the same co-cultivation conditions with *M. aeruginosa* were provided (Dunker et al., 2013). During these investigations, experimental conditions were chosen to exclude any kind of abiotic limitations of organisms by maintaining low cell densities. Nevertheless these two coccoid green algae showed completely different sensitivity in co-culture with *M. aeruginosa*. In this study the more general influence of cell wall structure on biotic interference mechanisms should be further investigated. For this purpose one additional coccoid green alga with tri-laminar/algaenan-containing cell wall and two additional species without these cell wall structure components were co-cultivated with *M. aeruginosa*.

## Materials and Methods

### Strains and Cell Cultivation

The growth of five algal strains (*Acutodesmus obliquus* (SAG 276-3a, formerly called: *Scenedesmus obliquus* (Turpin) Kützing, *S. acutus f. alternans*), *Desmodesmus armatus* (SAG 276-4 d, formerly called: *Scenedesmus quadricauda, Scenedesmus armatus* Chodat), *Chlorella vulgaris* (SAG 211-11b), *Oocystis marssonii* (SAG 257-1), and *Chloroidium saccharophilum* (SAG 211-9a, formerly called: *Chlorella saccharophila*)) was investigated in mono-culture and co-culture experiments with *M. aeruginosa* Kützing (SAG 48.60). All strains are from the SAG Culture Collection of Algae (Göttingen, Germany) and were cultivated in modified WC-Medium (Guillard and Lorenzen, 1972) in a light/dark cycle of 14/10 h at 20°C with a photon flux rate (Osram Lumilux, L36W/840, Cool White, Germany) of 130 μmol photons m^-2^ s^-1^ in 300 mL Erlenmeyer flasks. Modification of the medium was a change from Tris-buffer to 2 mM HEPES (2-[4-(2-hydroxyethyl)piperazin-1-yl]ethanesulfonic acid). As a pre-requisite of the experiments all cultures were semi-continuously cultured, at least 1 week in advance and were diluted approximately every third day to a biovolume of 10 mm^3^ L^-1^.

Samples were taken shortly after illumination start. The presented mean values are the means of at least three independent replicates. Significance was tested with the help of two-sided *T*-Tests.

### Cell Counting

The total cell numbers and cell diameter of uni-algal cultures were determined by means of a cell counter (Z2, Beckman Coulter, Drefeld, Germany). The fraction of cell numbers and the species-specific cell diameter of species in mixed cultures were measured by flow cytometry (Dunker et al., 2013). The biovolume of uni-algal cells was calculated from the median cell diameter (cell counter) assuming a spherical form.

In co-culture with *M. aeruginosa* half of the biovolume of mono-culture was inoculated. For comparison of biovolume development in mono-culture and co-culture the percentage deviation of half control biovolume was calculated.

### Flow Cytometry

A FacsAria II Sorp flow cytometer (Becton Dickinson, Heidelberg, Germany) equipped with a 70 μm nozzle was used for the estimation of the fraction of species-specific cell numbers and the calculation of cellular chl *a* concentration in the bi-algal cultures (Dunker et al., 2013). The Chl *a* fluorescence signals were used for the differentiation of the strains during co-cultivation. Chl *a* fluorescence was excited at 488 and 532 nm and the respective fluorescence emission was measured at 670 nm (LP) and 670 nm (BP 14 nm) which allowed to distinguish *M. aeruginosa* from green algae in two separate clusters due to their different pigmentation pattern (Dunker et al., 2013).

In general at least 5000 events were collected for data analysis, with only a limited number of exceptions. Flow cytometric data were analyzed with the FlowCore-package and visualized by FlowViz-package of R-software. Prior to analysis data were bi-exponentially transformed.

The accumulation of enzymatic resistant empty cell walls was measured with an ImageStream^®^^X^ Mk II flow cytometer (Merck Millipore, Darmstadt, Germany), which allows to identify single events of a cytogram by high throughput image analysis. Chl *a* fluorescence (Ex. 488 nm/Em. 702/85 nm) and cell area (μm^2^) were used to distinguish between empty cell walls and vital cells.

### Calcofluor Staining

**FIGURE 2:**
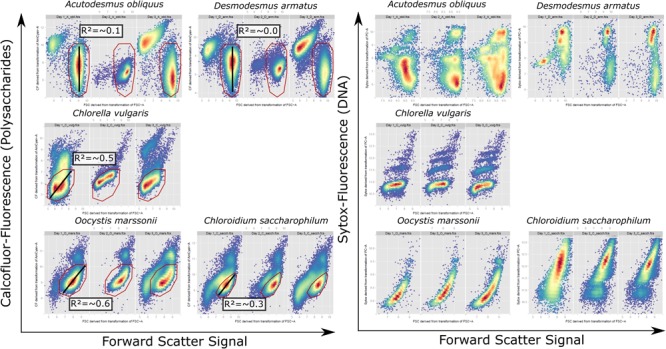
Cytometric pattern for FSC signal against Calcofluor fluorescence signal of vital cells and FSC-Signal against Sytox fluorescence signal of fixed cells for *A. obliquus, D. armatus, C. vulgaris, O. marssonii*, and *C. saccharophilum* from representative exemplary control cultures on day 1, 2, and 3. Bottom-right, high density population, was used for correlation of single cell values for FSC signal against Calcofluor fluorescence (red gate). Black line indicates the respective regression line and the mean regression coefficient for measurements over 1 week is given in the respective box.

Calcofluor White M2R (synonym: Fluorescence Brightener 28/ Fluostain 1) (Sigma-Aldrich) was used to detect polysaccharide cell wall components of green algae. 2.5 mg Calcofluor White M2R (hereafter called Calcofluor) was dissolved in 1 mL sterile distilled water, resulting in a 2.7 mM stock solution. This stock solution was prepared on the first day of each experiment and for each measurement 20 μL of this stock solution was pipetted to 1 mL of green algae sample, resulting in a final concentration of 53 μM Calcofluor. Samples were incubated for 10 min at room temperature. Afterward fluorescence was measured with the flow cytometer FacsAriaII (Becton Dickinson, Heidelberg, Germany) with an excitation of 405 nm and detection of emission signal from a 450/50 nm bandpass filter. For cell cycle analysis flow cytometric pattern from Forward Scatter-signal against Calcofluor fluorescence dotplots were used (**Figure 2**) (Dunker et al., 2017).

The Calcofluor/FSC-staining index was calculated to characterize coccoid species with respect to their staining susceptibility or resistance for Calofluor. Median Calcofluor-fluorescence data of each green algae species were corrected for cell size effects by division through median FSC-signal.

(1) $$\begin{array}{c} \frac{\mathrm{CF}}{\mathrm{FSC}}\;=\;\frac{{\mathrm{CF}}_{\mathrm{Median}\;\mathrm{of}\;\mathrm{population}}}{{\mathrm{FSC}}_{\mathrm{Median}\;\mathrm{of}\;\mathrm{population}}} \end{array}$$

Multidimensionality of growth was evaluated by a linear model of cellular values Calcofluor fluorescence against FSC signal for of high density regions (indicated by a line **Figure 2**), consisting of 1000–5000 single cell events.

### Crystal Violet Staining

Crystal Violet staining was prepared following the protocol of Zych et al. (2009). As modification, concentration of Crystal Violet was reduced to 0.2 g L^-1^ final concentration instead of 2 g L^-1^. Staining procedure was performed over 48 h instead of 24 h. Afterward three washing steps with WC-medium were performed, by centrifugation at 4000 *g* for 5 min. Three microscopic pictures (400x magnification) were taken to count stained cells (Supplementary Figure 1 and **Table 1**).

**Table 1:** Overview about investigated strains with respect to strain-number, taxonomic class and several indicators of cell wall resistance: percentage of Crystal Violet (CV), Ruthenium red (RR) and Toluidine blue O (TbO) stained cells, Calcofluor/Forward Scatter staining index (CF/FSC-SI), mean p-adjusted *R*-value of a linear model for high-density region over 5 days (CF/FSC-Corr.) and references of published algaenan-containing strains.

Strain	Class	Algaenan	CV stained cells	RR stained cells	TbO stained cells	CF/FSC-SI	CF/FSC-Corr.	Definition
*Acutodesmus obliquus* (SAG 276-3a)	Chlorophyceae	Yes [UTEX 1450 (Kodner et al., 2009); Strain 633 (Zych et al., 2009)]	25%	0%	0%	0.1 ± 0.0	0.0 ± 0.0	HR
*Desmodesmus armatus* (SAG 276-4 d)	Chlorophyceae	Yes (Allard and Templier, 2000)	16%	0%	30%	0.1 ± 0.0	0.1 ± 0.0	HR
*Chlorella vulgaris* (SAG 211-11b)	Trebouxiophyceae	No [Strain 136 (Zych et al., 2009)]	100%	8%	0%	0.7 ± 0.1	0.5 ± 0.1	MR
*Oocystis marssonii* (SAG 257-1)	Trebouxiophyceae	No (Robinson, 1981)	100%	0%	0%	2.3 ± 0.2	0.6 ± 0.1	LR
*Chloroidium saccharophilum* (SAG 211-9a)	Trebouxiophyceae	No (Burczyk et al., 1999; Zych et al., 2009)	100%	0%	0%	2.2 ± 0.1	0.4 ± 0.0	LR

*By consideration of all different indicators for cell wall resistance, the investigated strains were divided in highly resistant (HR), medium resistant (MR) and low resistant (LR) species.*

### Ruthenium Red Staining/Toluidine Blue Staining

According to the protocol of Soukup (2014) Ruthenium red and Toluidine blue O staining were separately prepared as a 0.01% solution (w/v). A 1:1 mixture for each dye with cell suspension was prepared. After 5 min staining time, a washing step with WC-medium as well as subsequent centrifugation with 4000 *g* for 5 min was performed.

### SYTOX Green Staining

For every sampling day of the experiment 1950 μL samples were fixed with 50 μL 25% glutardialdehyde and stored in a refrigerator at 4°C. For DNA-staining samples were centrifuged for 10 min at 4000 *g*. Subsequently supernatant was discarded and 960 μL WC-medium as well as 40 μL (containing 40 μg) RNAse (ThermoFisher) were added. The incubation of RNAse took 5 h and was performed in a ThermoMixer (Eppendorf) at 37°C. 20 μL of SYTOX green (25 μM stock solution) was added to RNAse treated samples and incubated overnight. Flow cytometric detection of SYTOX green was possible with the BD Facs Aria II and 488 nm (Exc.)/ 525/50 nm (Em.) excitation and emission.

#### FITC-Labeled Dextran Uptake (FD4 – 4 kDa Dextran)

The cell wall permeability for larger molecular weights was tested with FD4 (Sigma-Aldrich), a FITC-labeled dextran with a molecular size of 4 kDa. According to Ginzburg et al. (1999), cells were incubated with FD4 for a minimum of 1 h. As stock solution 4 mg FD4 mL^-1^ was prepared with Dulbecco‘s PBS (Biowest, Nuaillé, France). Final concentration was 1 mg FD4 mL^-1^ at a cell concentration of 1 × 10^6^ cells mL^-1^ Potential intracellular FITC-fluorescence was examined with an ImageStream^®^^X^ Mk II. Compensation for non-FD4-deriving fluorescence was performed with unstained cells. 1000–2000 cells were recorded for each treatment and species.

## Results

**FIGURE 3:**
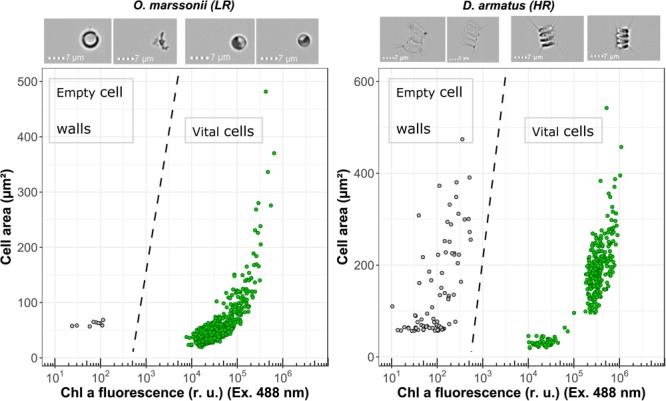
Flow cytometric measurements of Chl *a* fluorescence (Excitation 488 nm) and cell surface area for *O. marssonii* and *D. armatus*. Accumulation of enzymatic resistant empty cell walls is demonstrated by the population on the upper left-hand side. Representative microscopic pictures for the respective populations are placed on the top of the cytogram.

The investigated species were selected based on literature research for presence or absence of algaenan (**Table 1**). In addition, cell wall resistance for all investigated green algae was evaluated again by (a) cell inner staining with Crystal Violet, proposed by Zych et al. (2009), Ruthenium red and Toluidine blue O (b) by estimating the ratio of flow cytometrically measured Calcofluor-fluorescence to Forward-Scatter-signal and (c) exemplary flow cytometric detection of accumulated empty cell walls in the culture (**Figure 3**).

Crystal Violet is a common staining dye for Gram-positive and Gram-negative bacteria. Zych et al. (2009) suggested that the dye passively penetrates the cell wall of non-algaenan species, while algaenan-containing cell walls exclude the large molecules of Crystal Violet (MW 407.5 g mol^-1^). The authors noted that Crystal Violet remained in the non-algaenan containing algal cells, although rinsing was performed. *A. obliquus* and *D. armatus* showed highest staining resistance against Crystal Violet (Supplementary Figure 1 and **Table 1**). For *C. saccharophilum, C. vulgaris* and *O. marssonii* all cells were completely stained.

Ruthenium red is a hexavalent cation and binds to many polyanions, like, e.g., pectin in cell walls (Soukup, 2014). The procedure did not result in a clear positive staining for any of the strains (Supplementary Figure 2 and **Table 1**), but a small proportion of *C. vulgaris* cells could be stained with Ruthenium red, which would suggest a pectic cell wall component. Whereas in contrast a positive staining with a relatively high proportion of 30% for *D. armatus* with Toluidine blue O could be observed (Supplementary Figure 3 and **Table 1**), which is a metachromatic dye and the purple to pink color indicates a binding to pectin rich material (Soukup, 2014) or to anionic polysaccharides (Baudelet et al., 2017).

The dye “Calcofluor White” binds to different polysaccharide cell wall components which induces a violet fluorescence signal. For the calculation of CF/FSC staining index the Calcofluor-signal as median signal of the whole cell population was used. Since the species differ in cell size it was necessary to exclude size effects of Calcofluor-staining by calculating the ratio of Calcofluor fluorescence and FSC signal (indicating cell size). *O. marssonii* and *C. saccharophilum* show a high staining index for Calcofluor, *C. vulgaris* a medium and *A. obliquus* and *D. armatus* have the lowest staining CF/FSC staining indices (**Table 1**).

Cell wall permeability was further examined with a FITC-labeled dextran (FD4) and a respective molecular weight of 4 kDa. For none of the investigated species a significant intracellular uptake could be observed. In addition, image flow cytometry allows to identify accumulated empty cell walls in the sample as additional indication of resistant cell wall. It could be shown that for *D. armatus*, empty cell walls (recognizable by similar size of cells but no Chl *a* fluorescene and pictures) are present in the culture, while for *O. marssonii* no clearly identifiable empty cell walls could be found.

The rigidity of HR cell walls implies that cell size cannot increase their size multidimensionally. To test this multidimensionality aspect of cellular development within a population, Calcofluor fluorescence and FSC signals were measured by flow cytometry. Subsequently, Calcofluor fluorescence signal was correlated against FSC-signals for each cell within the sub-population of highest density region. The coefficient of correlation indicates either a continuous ratio of Calcofluor/FSC-Signal within this sub-population, no correlation or a non- linear ratio of both signals. For *A. obliquus* and *D. armatus* all cells of the sub-population marked by a polygon gate (**Figure 2**) have a similar FSC-signal but varying Calcofluor- and Sytox signals. This is an indication that cells in the gated subpopulation have similar cell size but various cell wall polysaccharide and DNA contents. In contrast, for *O. marssonii* and *C. saccharophilum* all cells show comparable ratios for Calcofluor-fluorescence/FSC-signal, meaning that smaller cells have lower polysaccharide and DNA-content per cell and larger cells higher polysaccharide and DNA contents per cell. For *C. vulgaris* the Calcofluor fluorescence/FSC cytogram is comparable to *O. marssonii* and *C. saccharophilum*, while Sytox fluorescence/ FSC-pattern are more similar to *A. obliquus* and *D. armatus*.

Growth characteristics of all species in mono- and co-culture were analyzed by different measures and levels. Median of FSC-signals, indicating cell size, and Chl *a*-fluorescence signal, indicating median Chl *a*-fluorescence per cell, were used as single cell characteristics. On a population level, increase of initial cell density and biovolume were considered. For co-cultures, biovolume data are derived from flow cytometric analysis. Interestingly all investigated species behave differently on single cell traits and on population level (**Table 2**).

**Table 2:** Overview about growth characteristics (The multiple of initial cell concentration, biovolume at day 1 and day 8 and percentage difference of measured to theoretical biovolume) and single cell properties (median Forward Scatter (FSC) Signal per cell and median Chl *a* fluorescence per cell) of all species under control and co-cultivation conditions, measured at day 8.

Species	Treatment	The multiple of			% difference to half of		
		initial cell density	Biovolume (mmł L^-1^)		control biovolume	FSC-Signal	Chl *a*-Signal
		Day 8	Day 1	Day 8		Day 8	Day 8
*Acutodesmus obliquus* (SAG 276-3a)	Control	22.5 ± 1.0	5.3 ± 0.5	115 ± 5		4234 ± 140	4519 ± 180(*)
	Mix (+ *M. aer.*)	19.1 ± 3.2	5.0 ± 0.5	73 ± 6	+26.9 ± 15.4	3869 ± 219	3969 ± 100
*Desmodesmus armatus* (SAG 276-4 d)	Control	19.1 ± 1.5	9.3 ± 0.7	156 ± 20		6341 ± 273	8520 ± 258
	Mix (+ *M. aer.*)	15.8 ± 2.4	5.9 ± 1.2	74 ± 16	–3.7 ± 24.9	6083 ± 216	9087 ± 584
*Chlorella vulgaris* (SAG 211-11b)	Control	24.5 ± 1.9	3.4 ± 0.4	84 ± 2		445 ± 50	751 ± 70
	Mix (+ *M. aer.*)	25.3 ± 2.0	2.3 ± 0.1	59 ± 4	+42.5 ± 12.9	460 ± 40	757 ± 47
*Oocystis marssonii* (SAG 257-1)	Control	15.4 ± 2.4	5.3 ± 0.7	82 ± 14		1821 ± 51	1886 ± 58
	Mix (+ *M. aer.*)	12.5 ± 1.0	2.3 ± 0.5	30 ± 6	–27.6 ± 8.0	1427 ± 166	1496 ± 165
*Chloroidium saccharophilum* (SAG 211-9a)	Control	14.9 ± 1.9(*)	7.1 ± 0.9	99 ± 12		2457 ± 224	2102 ± 327
	Mix (+ *M. aer.*)	9.5 ± 1.7	3.7 ± 1.1	27 ± 6	–45.4 ± 8.4	2254 ± 290	1846 ± 322
*Microcystis aeruginosa* (SAG 14.85)	Control	8.1 ± 1.0	21.1 ± 2.0	185 ± 12		1163 ± 92	430 ± 3
	Mix (+ *A. obl.*)	9.0 ± 0.3	9.5 ± 0.2	82 ± 2	–10.5 ± 7.0	1108 ± 24	453 ± 11
	Mix (+ *D. arm.*)	8.0 ± 1.0	9.2 ± 0.3	78 ± 4	–14.9 ± 9.3	1270 ± 106	474 ± 7(**)
	Mix (+ *C. vul.*)	5.7 ± 0.5	10.5 ± 0.4	57 ± 3	–38.7 ± 3.3	1120 ± 36	449 ± 15
	Mix (+ *O. mar.*)	10.1 ± 0.6	9.7 ± 0.5	118 ± 10	+28.7 ± 20.0	1496 ± 182	434 ± 12
	Mix (+ *C. sac.*)	9.8 ± 0.6	9.4 ± 0.9	114 ± 18	+23.7 ± 23.1	1559 ± 205	418 ± 13

*Significant results are indicated by a ^(∗)^ for *P* < 0.05 and by a ^(∗∗)^ for *P* < 0.01.*

**Table 2** shows, that *D. armatus* and *C. vulgaris* show comparable measured values for mono- and co-culture for the multiple of initial cell density and single cell properties (FSC-Signal and Chl *a*-Signal). For cultures of *C. saccharophilum* and *O. marssonii* this picture is different. While the multiple of initial cell density of *C. saccharophilum* in co-culture with *M. aeruginosa* is significantly lower than in mono-culture, for *O. marssonii* single cell properties tend to be lower than in mono-culture. For these two species biovolume on day 8 was 30–40% lower than expected from mono-culture controls (Supplementary Figure 4). In contrast, biovolume of *D. armatus* and *C. vulgaris* in co—culture was similar (*D. armatus*) or higher than expected from mono-culture (*C. vulgaris*). For *A. obliquus* biovolume on day 8 was higher in co-culture than expected from control. In contrast, Chl *a*-signal per cell was significantly lower than under control conditions.

Interestingly growth was not only affected in green algae, but also growth of *M. aeruginosa* partially deviated from control in some co-cultures. For example the multiple of initial cell density in co-culture was lower when *M. aeruginosa* was co-cultivated with *C. vulgaris* and higher with *O. marssonii* and *C. saccharophilum*. Also FSC signal of *M. aeruginosa* was higher than in mono-culture when it was co-cultured with *O. marssonii* and *C. saccharophilum*. Chl *a* signal was higher in co-culture with *A. obliquus* and significantly higher in co-culture with *D. armatus*.

As an additional measure of difference between mono- and co- culture growth performance, the percentage deviation of half control biovolume was calculated. From these values it is conspicuous that biovolume of *D. armatus* was the only one not influenced by co-cultivation with *M. aeruginosa*. For *A. obliquus* and *C. vulgaris* a higher percentage of biovolume in comparison to mono-culture control was found. In contrast to this better growth performance on the basis of higher biovolume, in co-cultures of *O. marssonii* and *C. saccharophilum* a decrease of growth in co-culture with *M. aeruginosa* was obvious. *M. aeruginosa* in contrast was positively influenced in co-culture with *O. marssonii* and *C. saccharophilum* while a negative effect occurred in co-culture with *A. obliquus, D. armatus*, and *C. vulgaris*. Co-cultivation with *C. vulgaris* led to strong decline of *M. aeruginosa* growth.

## Discussion

In this work cell wall structure and its influence on biotic interference interaction was investigated for the first time. The cell wall structure is an important ecological trait because cell wall properties determine the extent of contact to surrounding environment. Therefore, the cell wall is exposed to many kinds of stress, like viral, bacterial or fungal attack (Largeau and De Leeuw, 1995; Versteegh and Blokker, 2004), where, it seems to be an advantageous feature to have a HR-cell wall in environments with a high microbial density and therefore high probability of extracellular enzymatic attack. However, there must be a trade-off between resistance and permeability of the cell wall, explaining the evolutionary established lack of resistant compounds in some species. One possible explanation is the difficulty of growth and autospore release with an algaenan-containing cell wall (**Figure 1**). A highly rigid cell wall aggravates a continuous multidimensional growth in cell size. Diatoms also possess a rigid cell wall, but the so-called girdle band with lower rigidity allows reproduction and unidimensional increase in cell size during auxospore formation (Hecky et al., 1973). We tested the hypothesis that coccoid green algae with a highly resistant, algaenan-containing cell wall are less sensitive in co-cultivation with *M. aeruginosa* than species with a LR-cell wall.

### Species Selection

The selection of species by presence or absence of algaenan was based on a literature survey (**Table 1**). To proof the selection of strains, Crystal Violet, Calcofluor, Ruthenium red, Toluidine blue O and FD4-staining were performed to characterize the investigated coccoid strains with respect to cell wall properties. These findings support the work of Kodner et al. (2009) and Zych et al. (2009) who showed algaenan as present in the species *A. obliquus*. Also for *D. armatus* the presence of algaenan was demonstrated before (Allard and Templier, 2000). For *C. vulgaris* (Strain 136) (Zych et al., 2009) and *C. saccharophilum* (Yamada and Sakaguchi, 1982; Burczyk et al., 1999; Zych et al., 2009) it was shown that they contained no algaenan and were sensitive for Crystal Violet staining (Zych et al., 2009). For *O. marssonii* no literature data were available with respect to the occurrence of algaenan in this alga. An indirect hint was found, where it was stated, that the cell wall of *Oocystis solitaria* has homogenous structure and not an electron-dense layer comparable to species of *Chlorella* and *Scenedesmus* (Robinson, 1981).

### Crystal Violet and Calcofluor Staining

In summary *A. obliquus* and *D. armatus* showed high resistance against staining with either Crystal Violet or Calcofluor (low CF/FSC-Staining Index). In addition, both species have low correlation values in flow cytometric pattern of gated high-density sub-population (low correlation between Calcofluor fluorescence and FSC).

In contrast *C. saccharophilum* and *O. marssonii* were stained with Crystal Violet and Calcofluor, indicated by a high CF/FSC-staining index. This is in accordance to Yamada and Sakaguchi (1982), who reported a high Calcofluor fluorescence intensity for *C. saccharophilum*. The correlation between CF and FSC signals was positive with a high correlation value. In contrast *C. vulgaris* had a different Calcofluor/FSC-ratio and flow cytometric pattern. According to Burczyk et al. (1999) within non-algaenan coccoid algae, two groups can be differentiated, one group with high and one with low glucosamine content in the outer cell wall. It is hypothesized that the investigated *C. vulgaris* strain (SAG 211-11b) belongs to the group of high glucosamine and non-algaenan-containing coccoid green algae. Besides algaenan also glucosamines are discussed as main constituent of rigid cell walls (Takeda, 1993; Burczyk et al., 1999). Takeda and Hirokawa (1978) showed that the maximum yield of glucosamine as rigid wall constituent can be about 50% for *Chlorella ellipsoidea*. From the ratio of Calcofluor-staining to FSC-signal the proportion of rigidity determining constituents like algaenan and glucosamine can be indirectly assessed.

Pectin containing cell wall layer could be assumed for *C. vulgaris* and *D. armatus*, where potential function could be that they are involved in cell wall expansion (Eder and Lütz-Meindl, 2010), adsorbents of chemicals, e.g., metal ions (Macfie and Welbourn, 2000) or can be a structural component to hold daughter cells together during cell division (Popper et al., 2011). The staining of Crystal Violet, Calcofluor, Ruthenium red and Toluidine blue O, as well as flow cytometric patterns allow to assign the investigated species to three different groups: low resistant (LR) group (*O. marssonii* and *C. saccharophilum*), medium resistant (MR) group (*C. vulgaris*) and highly resistant (HR) group (*A. obliquus* and *D. armatus*).

It is hypothesized that the multidimensionality of cellular growth is aggravated in HR cell wall structure-type species. It could be shown that flow cytometric monitoring during the growth process can reveal the multidimensionality or continuity of cell properties within a population. Over 5 days the correlation of Calcofluor-fluorescence to FSC-signal values for high-density regions of single cells is continuously low in species with rigid and high in species with non-algaenan cell wall. The multidimensionality aspect of cellular growth cannot be only shown by Calcofluor/FSC signals, but also for DNA-staining by Sytox Green which reveals similar pattern for the investigated species. *A. obliquus* and *D. armatus* have a comparable cell size but different DNA contents, which was also described by Zachleder et al. (2002). In contrast, *O. marssonii* and *C. saccharophilum* show a strong positive correlation between FSC signal and DNA-content.

Staining with different dyes indicates an accessibility or resistance to dyes. Therefore, it is on the one hand possible to use the flow cytometric Calcofluor/FSC-staining indices to assess different cell wall structure types of coccoid green algae which reveal a probability of susceptibility against enzymatic attack or resistance. It is conceivable that flow cytometric measurements can be used as additional taxonomic markers for different genera or classes as supplement to morphological and molecular identification tools of coccoid species (Krienitz and Bock, 2012). On the other hand great care has to be taken by quantitatively comparing fluorescent dyes when using different coccoid species.

For the second step of this study, species with these three different cell wall types were tested for interference interaction with *M. aeruginosa*.

As evidence for the hypothesis of cell wall structure influence on interference competition, it was shown that during co-cultivation with *M. aeruginosa* growth was negatively affected the most in low resistant species *O. marssonii* and *C. saccharophilum*, while HR-species *D. armatus* showed non-influenced growth or even better growth for *A. obliquus* on the base of percentage biovolume deviation from control. *C. vulgaris*, defined as MR-species, showed a substantial improvement of growth in co-culture in comparison to mono-culture control. *C. vulgaris* also influenced *M. aeruginosa* negatively the most.

**FIGURE 4:**
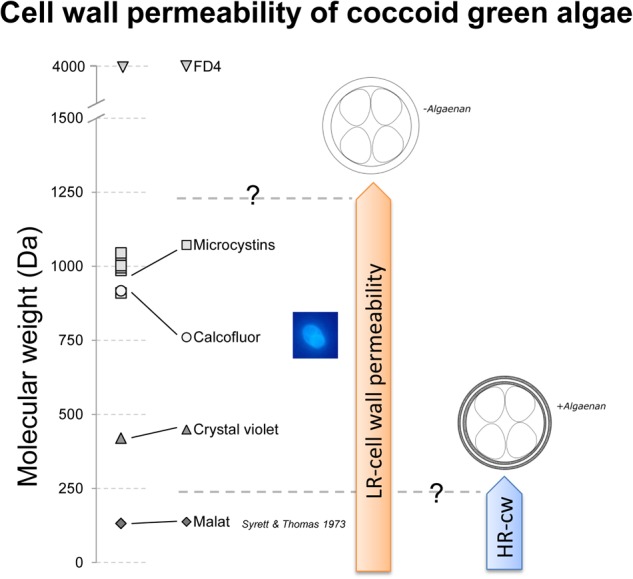
Schematic drawing of cell wall permeability of low resistant (LR)- and highly resistant (HR)-cell wall coccoid green algae for malate, Crystal Violet, Calcofluor, microcystins and FITC-labeled dextran (FD4 – 4 kDa). Malate, Crystal Violet, and Calcofluor were shown to pass the cell wall for LR-cell walls, but only malate was demonstrated by Syrett and Thomas (1973) to pass the cell wall of HR-cell wall types. Microcystins, relevant for biotic interference interaction have a relevant molecular weight to be permeable to LR-cell wall types.

The mode of action of growth inhibition in LR-cell wall species is not completely clear yet, but it would be necessary to know, if the cell wall acts as a structural barrier or as sequestering mechanisms, like it was shown for pectins and heavy metal adsorption in plant cells and algae (Button and Hostetter, 1977; Parrotta et al., 2015). The most prominent toxins of *M. aeruginosa* are microcystins, inhibiting protein phosphatases. The molecular weight of microcystins varies in the range of 909 to 1115 Dalton (Bláha et al., 2009; Walker, 2014), where the HR-cell wall could definitively act as a structural barrier (**Figure 4**). Jungmann (1995) describes another compound produced by *Microcystis flos-aquae*, toxic for *Daphnia*, which was most toxic in the size fraction between 1 and 3.3 kDa. In addition, Sukenik et al. (2002) describe a carbonic anhydrase inhibiting toxic compound excreted by *Microcystis sp.* with a size of <5 kDa. Both substances, as well as microcystins could be candidates for growth inhibition of the LR-cell wall species. In **Figure 4** Calcofluor is listed with a comparable molecular weight like microcystins. Although it is described that CF is a staining dye for cell surface carbohydrates, we could see a potentially intracellular staining of daughter cells (image of a daughter cell containing *O. marsonii* cell), which may indicate a permeability for this molecular weight fraction and a sensitivity toward microcystins. The cell wall permeability study with FD4 revealed that 4 kDa is already a limit for investigated LR-cells. Unfortunately the 4 kDa FITC-labeled dextran was the lowest molecular weight fraction we could find, which is why the maximum size of cell wall permeability could not be determined more precisely.

In Dunker et al. (2017) strong evidence was shown that the interference mechanism between *O. marssonii* and *M. aeruginosa* is based on cell-cell-contact. If this is the case also for growth reduction in *C. saccharophilum* and not in *D. armatus, A. obliquus* and *C. vulgaris*, cell wall structure seems to be the basic requirement of cell-cell-aggregation. Probably the cell wall of *D. armatus* and *A. obliquus* is used as a disposable protective cover. It is known that algaenan-containing cell walls accumulate over time in the culture medium, because they will not be quickly degraded (Burczyk et al., 1999). They contain a number of enzymatic activities, e.g., phosphatases, esterases and various glycosidases remaining long time in the liberated cell wall (Burczyk and Loos, 1995). These empty cell walls can be detected as pink fraction after successive centrifugation steps. In this study, image cytometry allowed to detect a high amount of accumulated empty cell walls in a sample of *D. armatus* culture in contrast to *O. marssonii* culture, where no accumulated cell walls could be detected (**Figure 3**). The energy costs for synthesis of protective HR-cell walls is expected higher than for LR-cell walls because of suggested synthesis through acetate/malate pathway, leading to high energy requiring fatty acids (Versteegh and Blokker, 2004). A first hint for energy demand can be derived from calculations how many absorbed photons are used for biomass formation – the quantum efficiency of growth. Dunker et al. (2013) calculated this quantum efficiency for *O. marssonii* and *A. obliquus* when grown under same culture conditions. With 7 mol photons (g dry weight)^-1^ *O. marssonii* was more efficient to form biomass than *A. obliquus*, which needed 9 mol photons (g dry weight)^-1^. Due to different general macromolecular composition of the two species, this quantum efficiency does not only reveal cell wall costs, but general metabolic costs. It is conceivable that, depending on growth reduction induced by interference interaction, this cost for HR-cell wall could be amortized in a short time period. Besides algaenan as HR-cell wall constituent, also glucosamine could improve the resistance of cell wall, which is assumed to be energy requiring, too. Glucosamine also could help to withstand biotic interference interaction, like it could be shown for *C. vulgaris*, although the resistance of *C. vulgaris* against Crystal Violet was not comparable to *A. obliquus* and *D. armatus*.

A diversification of coccoid cell wall types could be partly based on interference competition. In general, trade-offs between metabolic costs, cell development and protection efficiency against attacks by virus, bacteria, fungi or even other phytoplankton species could be a possible explanation for several evolutionary solutions and heterogeneous pattern of cell wall ultrastructure.

## Conclusion

As analog to gram-staining for bacteria it was shown that Crystal Violet can be used to differentiate cell wall structure-types, like it was also shown by Zych et al. (2009). Based on Crystal Violet, Calcofluor, Ruthenium red and Toluidine blue O-staining as well as from cytometric pattern three different cell wall structure types were identified (LR, MR and HR) and tested for interference competition with *M. aeruginosa*. Both low resistant species were affected and one medium as well as both highly resistant species were not affected in co-culture with *M. aeruginosa*. The data of this study show evidence for the important role of cell wall ultrastructure not only in protection against chemical compounds, virus, bacterial, fungal, zooplankton gut enzyme attack but also for interference interaction with phytoplankton species. There seems to be trade-offs between resistance, cell wall development and energy requirement if a HR-or LR-cell wall is preferred in a given environment. In future it would be an interesting task to investigate if co-evolution of cyanobacteria and coccoid green algae may have diversification effects. Furthermore it is necessary to prove the general applicability of these findings with more species.

## Author Contributions

SD: Main author of the study and performance of all experimental work. CW: Substantial contribution to the conception of the work and final approval of the version to be published.

## Conflict of Interest Statement

The authors declare that the research was conducted in the absence of any commercial or financial relationships that could be construed as a potential conflict of interest.

## Abbreviations

Chl *a*: Chlorophyll *a*
FSC-signal: Forward Scattering-Signal
HR-cell wall: highly resistant cell wall
LR-cell wall: low resistant cell wall
MR-cell wall: medium resistant cell wall
TLS: tri-laminar structure/sheath
