# Supplementary material for: Cell Wall Structure of Coccoid Green Algae as an Important Trade-Off Between Biotic Interference Mechanisms and Multidimensional Cell Growth
Source: Front Microbiol. 2018 Apr 13;9:719. doi: 10.3389/fmicb.2018.00719 (PMC5908957; doi:10.3389/fmicb.2018.00719)
Supplement: Supplementary file 1 [file Data_Sheet_1.DOCX]

**Supplemental figures**

Supplemental figure 1: Microscopic images of 48h-crystalline violet stained cells from two highly resistant (HR) species *A. obliquus* and *D. armatus*, one medium resistant (MR) strain *C. vulgaris*, as well as two low resistant (LR) species *O. marssonii* and *C. saccharophilum*.

Supplemental figure 2: Microscopic images of Ruthenium red stained cells from two highly resistant (HR) species *A. obliquus* and *D. armatus*, one medium resistant (MR) strain *C. vulgaris*, as well as two low resistant (LR) species *O. marssonii* and *C. saccharophilum*.

Supplemental figure 3: Microscopic images of Toluidine blue O stained cells from two highly resistant (HR) species *A. obliquus* and *D. armatus*, one medium resistant (MR) strain *C. vulgaris*, as well as two low resistant (LR) species *O. marssonii* and *C. saccharophilum*.

Supplemental figure 4: Differences of biovolume for the respective species in mixed cultures, expressed as percentage deviation from half of control biovolume. The first 5 bars show *A. obliquus*, *D. armatus*, *C. vulgaris*, *O. marssonii* and *C. saccharophilum* when cultivated with *M. aeruginosa* (*+M. aerug*.), the last 5 bars show *M. aeruginosa* when grown with *A. obliquus*, *D. armatus*, *C. vulgaris*, *O. marssonii* and *C. saccharophilum. (+ A. obliqu., +D. armat., +C. vulg., +O. mars., +C. sacch.).*

Supplemental figure 1

Supplemental figure 2


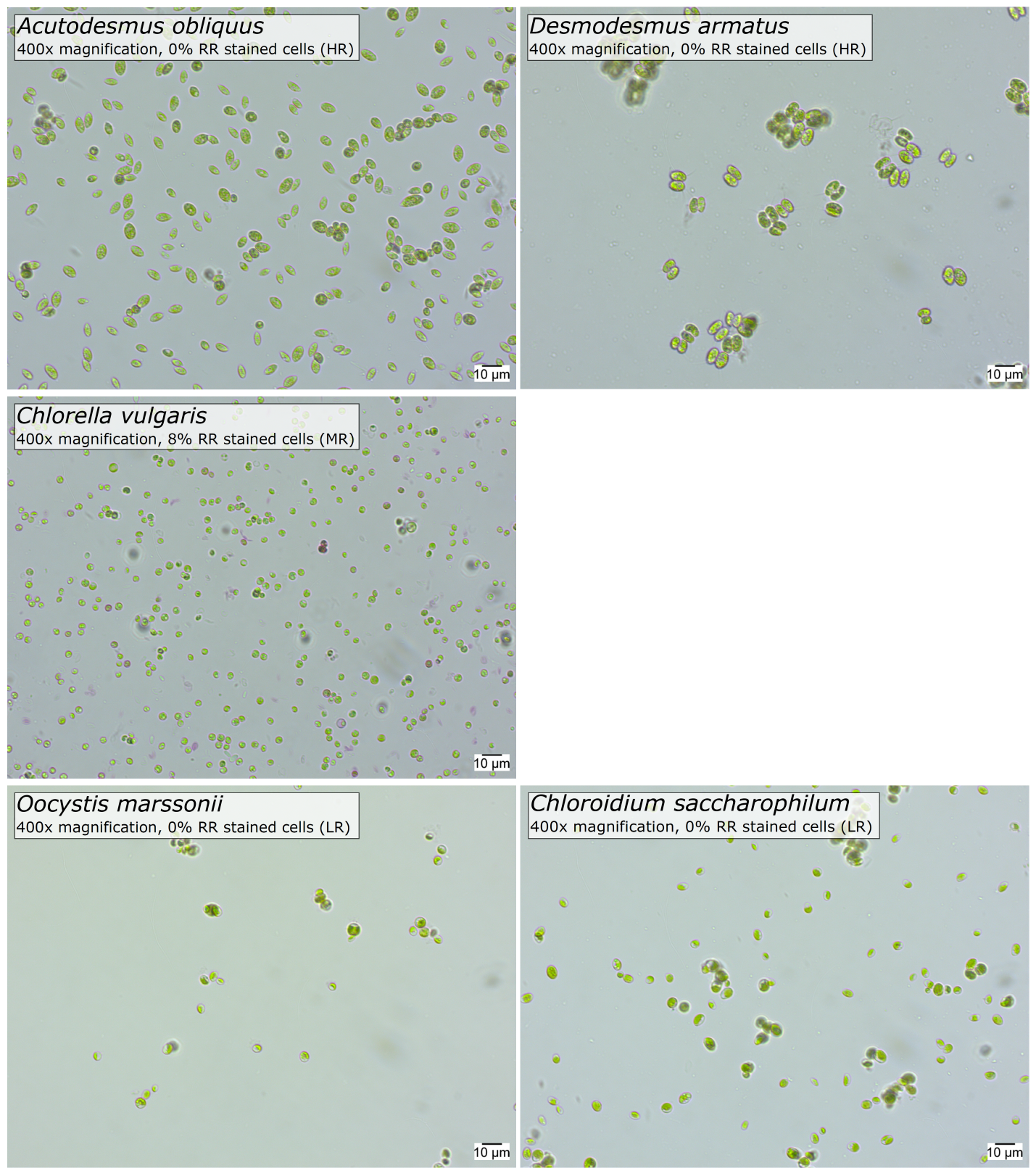


Supplemental figure 3


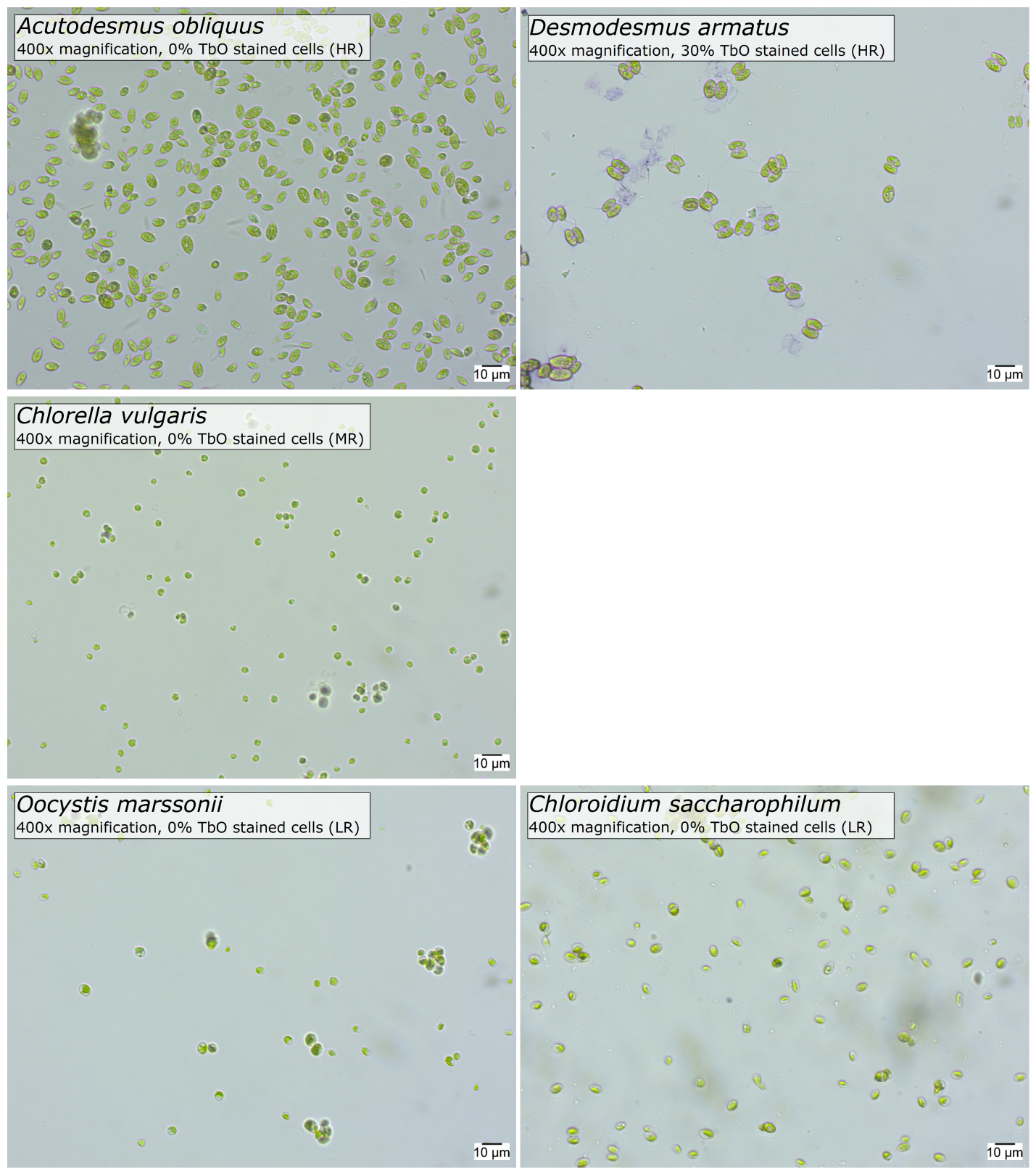


Supplemental figure 4
